# The Sensing Properties of Single Y-Doped SnO_2_ Nanobelt Device to Acetone

**DOI:** 10.1186/s11671-016-1685-1

**Published:** 2016-10-21

**Authors:** Xinmin Li, Yingkai Liu, Shuanghui Li, Jieqing Huang, Yuemei Wu, Dapeng Yu

**Affiliations:** 1 Key Laboratory of Yunnan Higher Education Institutes for Optoelectronic Information and Technology, Kunming, 650500 People’s Republic of China; 2 Key Laboratory of Yunnan Normal University for Photoelectric Materials & Device, Kunming, 650500 People’s Republic of China; 3 Institute of Physics and Electronic Information, Yunnan Normal University, Kunming, 650500 People’s Republic of China; 4 Department of Physics, State Key Laboratory for Mesoscopic Physics, Peking University, Beijing, 100871 People’s Republic of China

**Keywords:** SnO_2_ nanobelts, Y^3+^ doping, Gas sensor, Optical properties, Acetone

## Abstract

Pure SnO_2_ and Y-doped SnO_2_ nanobelts were prepared by thermal evaporation at 1350 °C in the presence of Ar carrier gas (30 sccm). The samples were characterized by scanning electron microscope (SEM), X-ray diffraction (XRD), energy dispersion spectrometer (EDS), X-ray photoelectron spectrometer (XPS), UV-Vis absorption spectroscopy, Raman spectroscopy, and Fourier transform infrared spectrum (FTIR). The sensing properties of the devices based on a single SnO_2_ nanobelt and Y-doped SnO_2_ nanobelt were explored to acetone, ethanol, and ethanediol. It reveals that the sensitivity of single Y-doped SnO_2_ nanobelt device is 11.4 to 100 ppm of acetone at 210 °C, which is the highest response among the three tested VOC gases. Y^3+^ ions improve the sensitivity of SnO_2_ sensor and have an influence on the optical properties of Y-doped SnO_2_ nanobelts.

**Electronic supplementary material:**

The online version of this article (doi:10.1186/s11671-016-1685-1) contains supplementary material, which is available to authorized users.

## Background

With the development of science and technology as well as people’s increasing concerns for the environment, considerable attentions are paid to efficiently and precisely detect and supervise flammable, explosive, or poisonous gases [1].

As a transparent n-type semiconductor with a band gap of 3.6 eV, SnO_2_ can be used as photoelectric devices, sensors, catalysts, and other functional materials [2]. Due to the unique physicochemical properties of SnO_2_ and enhanced sensing properties of nanostructured materials, quasi-one-dimensional (1D) SnO_2_ nanomaterials are being widely studied [3]. Various methods were developed to synthesize nanostructured SnO_2_ materials, such as the sol-gel method, liquid precursor method [4], electroplating tin thermal oxidation method [5], and chemical vapor deposition (CVD) method [6]. Therefore, synthesis of 1D nanostructured SnO_2_ materials has made great achievements [7, 8]. SnO_2_ with various morphologies such as nanoparticle, nanowire, nanosilk, nano-sawtooth, nanobelt, or nanotube are obtained by the abovementioned methods [9–11], which can be used as building blocks for functional devices [12, 13]. Inherent small size effect and surface effect of nanomaterials make SnO_2_ possess particular physicochemical properties, which are beneficial for gas sensors and solar cells [14–17].

From the point view of pollution, acetone (a common reagent used widely in industries and labs) is harmful to human health. It is extensively used to dissolve plastic, purify paraffin, and dehydrate tissues in pharmaceutics [18]. Inhalation of acetone causes headache, fatigue, and even narcosis and harmfulness to the nerve system. Hence, it is necessary to monitor acetone concentration in the environment for health and safety purposes in the factory [19].

In this work, we undertake the study on the fabrication and characterization of the devices based on a single SnO_2_ nanobelt (NB)/Y-SnO_2_. After that, we systematically investigate the sensing properties of single SnO_2_ NB/Y-SnO_2_ NB device. Based on it, the influence of Y elements on the sensing properties of SnO_2_ NB is discussed.

## Methods

### Synthesis of Y-Doped SnO_2_ NBs

Y-doped SnO_2_ NBs (hereafter denoted as “Y-SnO_2_ NBs”) were prepared by thermal evaporation technique. For synthesis of Y-SnO_2_ NBs, SnO_2_ powders with a purity of 99.99 % were mixed with Y powders (Yttrium (III) acetate tetrahydrate 99.99 %) in the weight ratio of 20:1 and then put into a ceramic boat. The boat was placed in the center of the alundum tube, which was installed in a high-temperature furnace. A silicon substrate coated with about 10-nm-thick Au film was put in the alundum tube with a distance of 10 cm from the ceramic boat and then the tube was cleaned several times by argon gas. The temperature of the furnace was heated up to 1350 °C at a rate of 15 °C/min and was kept for 2 h. Ar gas was flowed at 30 sccm, and the pressure inside the tube was maintained to 112.5 Torr during the whole experiment. The deposited samples were taken out as the furnace was naturally cooled to room temperature.

The morphology, microstructures, and composition of Y-SnO_2_ NBs were characterized by X-ray diffraction (XRD), scanning electron microscopy (SEM), energy dispersive X-ray spectroscopy (EDS), transmission electron microscopy (TEM), UV-Vis absorption spectra, Raman spectra, Fourier transform infrared spectrum (FTIR), and high-resolution transmission electron microscopy (HRTEM).

**Fig. 1:**
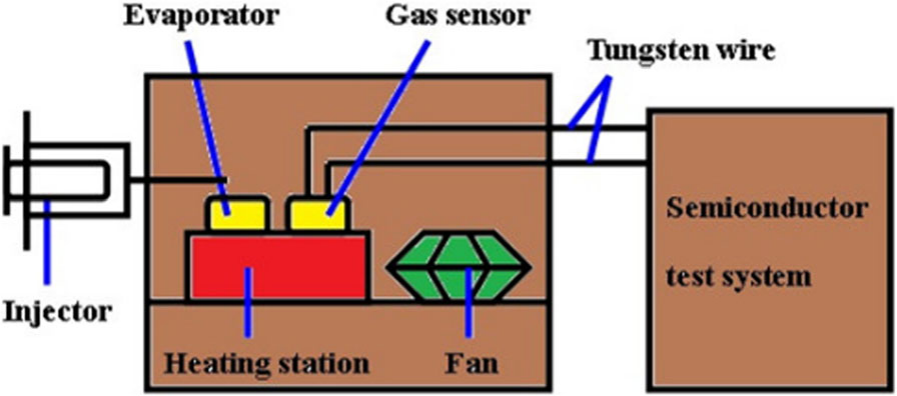
The schematic diagram of the test system

The device was fabricated as follows: for fabrication of a single nanobelt sensor, SnO_2_ NBs and Y-SnO_2_ NBs were scratched by tweezers and some products were dispersed in ethanol, respectively. The resulting suspension was dropped onto the silicon substrate with a 500-nm-thick SnO_2_ layer. After the ethanol evaporated completely, Ti (8 nm) and Au (100 nm) electrodes were deposited by dual-ion beam deposition system (LDJ-2a-F100-100 series) with the assistance of a meshgrid mask composed of tungsten wires (10 μm in diameter). The vacuum was kept at 2.2 × 10^−2^ Pa in the whole process, and Ar was flowed at 10 mA/cm^2^. The sensing test system is illustrated in Fig. 1. The measurements were conducted in a hermetic stainless steel vessel (20 L). Then, the sensor linked with the semiconductor test system by tungsten wires was put on a heating station. The target liquid would be injected into an evaporator to quickly evaporate, and a fan was used to produce homogeneous atmosphere in the chamber. Finally, the gas sensing performance of the devices was measured by Keithley 4200.

## Results and Discussion

### Morphology and Structure

**Fig. 2:**
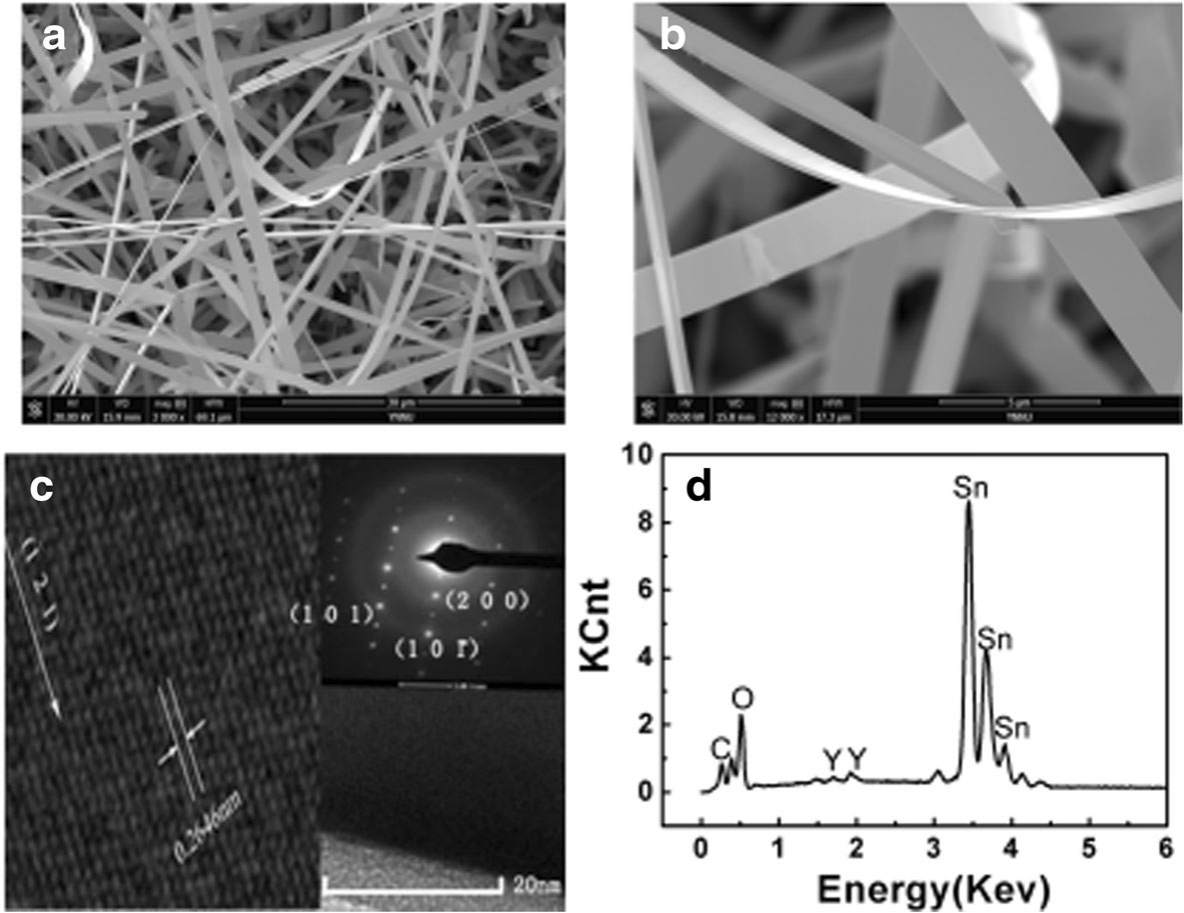
Low-magnification SEM image (**a**). High-magnification SEM micrograph of Y-SnO_2_ nanobelts (**b**). HRTEM image, the *inset*: SAED pattern (**c**). EDS pattern of Y-SnO_2_ NBs (**d**)

The morphology of the Y-SnO_2_ was observed by scanning electron microscopy, as shown in Fig. 2a. The products are nanobelts, which are randomly stacked together; many filamentous structures were presented. A high-magnification SEM image in Fig. 2b reveals that the obtained NBs are of smooth surfaces with a thickness of 30–50 nm and length up to 40 μm. HRTEM image of a Y-SnO_2_ NB is displayed in Fig. 2c and the interplanar spacing is 0.2646 nm, and the growth direction is along [−121]. The EDS pattern of a Y-SnO_2_ nanobelt was shown in Fig. 2d. The elements of C, O, Sn, and Y were observed in EDS. It confirms the existence of Y^3+^ in the nanobelt and the content of Y is 0.86 wt.%.

**Fig. 3:**
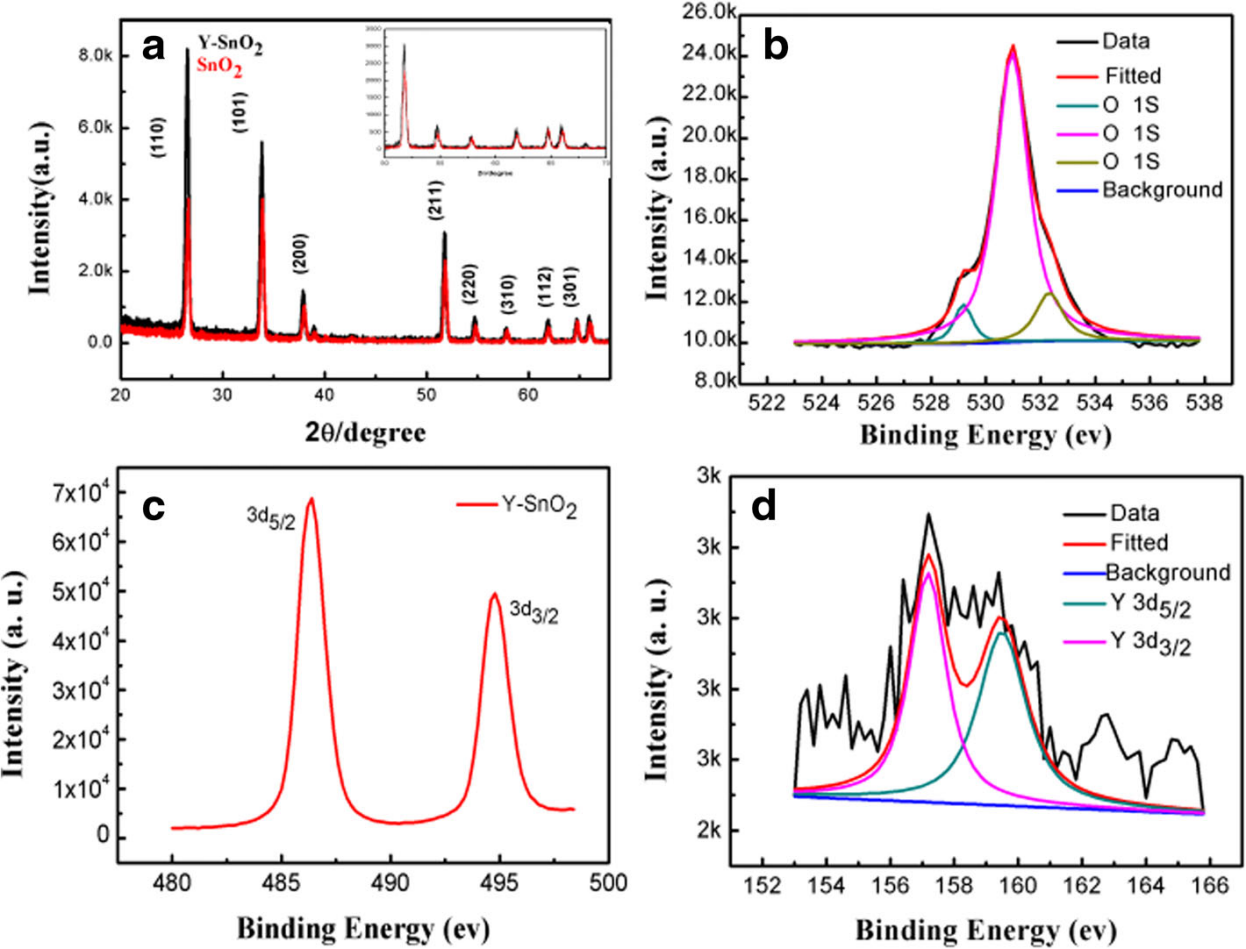
XRD patterns of Y-SnO_2_ and undoped SnO_2_ (**a**). XPS spectrum of superposed O (1s) (**b**), Sn (3d) (**c**), and Y (3d) (**d**), respectively

The XRD and XPS pattern of Y-SnO_2_ NBs and undoped counterparts are presented in Fig. 3. It is found that all well-defined diffraction peaks can be indexed as the tetragonal structure SnO_2_ with lattice parameters *a* = *b* = 0.4738 nm, *c* = 0.3188 nm (JCPDS card no. 71-0467). The positions of the diffraction peaks (2*θ* = 26.5°, 33.7°, 37.8°, 51.8°, 54.8°, 57.7°, 61.8°, and 64.6°) matched with the crystal plane ((110), (101), (200), (211), (220), (310), (112), and (301), respectively). No other impurities are detected, indicating that the doping of Y element does not cause the change of crystal structures. Comparing with XRD of pure SnO_2_, the diffraction peaks of Y-SnO_2_ NBs corresponding to (211), (220), (310), (112), and (301) lattice planes shift towards the low-angle direction, as shown in the inset of Fig. 3a. The reason is that Y^3+^ ions (radius 89 pm) replace the position of Sn^4+^ (radius 69 pm); the lattice parameters of Y-SnO_2_ NBs become larger than those of pure SnO_2_, revealing that Y^3+^ ions have been doped into the lattices of SnO_2_.

The XPS spectra for the binding energy of Sn (3d), O (1s), and Y (3d) electrons are also provided to demonstrate the existence of Y^3+^ ions. The deconvolution of the O (1s) peak shows three Gaussian peaks, centered at 529.3, 530.98, and 532.5 eV, respectively (displayed in Fig. 3b). The peak at the low-binding energy can be attributed to the lattice oxygen in SnO_2_ and the high-binding energy related to the chemisorbed oxygen species. The Sn (3d) peak shows two peaks located at the binding energies of 486.3 eV Sn (3d_5/2_) and 494.7 eV of Sn (3d_3/2_), as shown in Fig. 3c. The separation distance between the two peaks is 8.4 eV, which corresponds to the Sn standard spectrum, indicating the formation of Sn^4+^ oxidation state in the SnO_2_ nanobelts [20]. The Y (3d) can be separated into two peaks; the peaks at 157.2 and 159.98 eV belong to the binding energies of Y (3d_5/2_) and Y (3d_3/2_), respectively, as displayed in Fig. 3d. These results are in good agreement with those of XRD and EDS. Therefore, it is confirmed that Y^3+^ ions are doped into SnO_2_ nanobelts successfully.

### Optical Properties

**Fig. 4:**
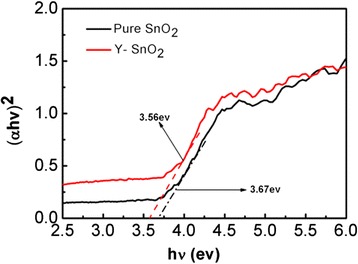
UV-Vis absorption spectra of Y-SnO_2_ nanobelts and pure SnO_2_ nanobelts. The corresponding (αhν)^2^ versus hν curves

The UV-Vis absorption spectra of Y-SnO_2_ NBs and pure SnO_2_ NBs are presented in Fig. 4. The energy band gap was determined to be 3.56 eV for the Y-SnO_2_ NBs and 3.67 eV for the pure SnO_2_ ones, respectively. Compared with that of pure SnO_2_, the UV-Vis absorption peak of Y-SnO_2_ NBs redshifts after doping. Impurity energy levels in the band gap will change into impurity bands due to the interaction between the impurity ions and the based lattices [21, 22].

**Fig. 5:**
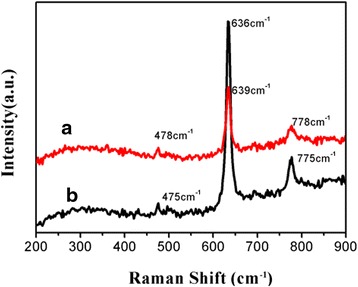
Raman spectra of Y-SnO_2_ NBs (*a*) and pure SnO_2_ NBs (*b*)

Figure 5 shows the Raman spectra of pure and Y-SnO_2_ NBs measured at room temperature. It is seen that three peaks of Y-SnO_2_ NBs can be observed in Fig. 5a, which are located at 478, 639, and 778 cm^−1^, respectively. In the meantime, we also found that the intensity of the Raman peak centered at 300 cm^−1^ for Y-SnO_2_ NBs is slightly higher than that of its counterpart. Compared with pure SnO_2_ in Fig. 5b, its spectrum has not changed much after doping. However, it is noted that the Raman peaks of Y-SnO_2_ NBs happen to redshift by the quantitative analysis. The intensity difference of Raman peaks for the pure SnO_2_ NBs and Y-SnO_2_ NBs can be attributed to the crystalline sizes of the samples [23].

### Sensing Properties

**Fig. 6:**
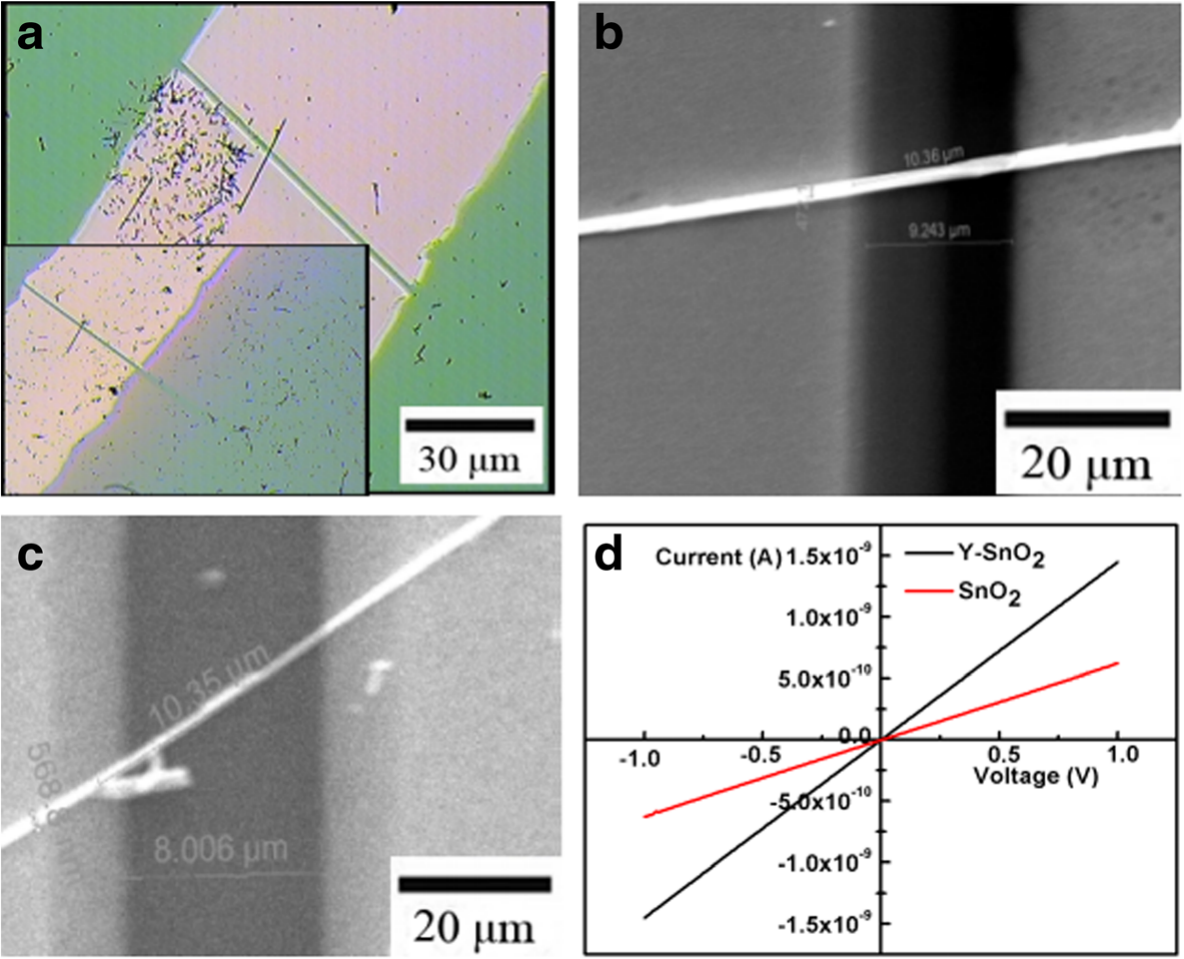
The optical microscopic image of the pure SnO_2_ NB. *Inset*: the optical microscopic image of the Y-SnO_2_ NB (**a**); SEM image of a single SnO_2_ NB (**b**); SEM image of a single Y-SnO_2_ NB (**c**); I–V curves of the pure SnO_2_ and Y-SnO_2_ NB devices (**d**)

Figure 6a shows a typical optical microscope image of the obtained Y-SnO_2_ NB and pure SnO_2_ NB device, which is composed of an individual nanobelt and Au electrodes. Figure 6b, c presents the SEM image of Fig. 6a, which is used for all gas sensing measurements. The thickness of Y-SnO_2_ NB and pure SnO_2_ NB is about 50 nm. The length and width of the pure SnO_2_ NB and Y-SnO_2_ NB are about 10.36 and 10.35 μm and 472.1 and 568.8 nm, respectively. The calculated results confirmed that the surface ratio of Y-SnO_2_ NB to pure SnO_2_ NB is 1.1 (see Additional file 1 for more details). Figure 6d shows the I–V curves of pure SnO_2_ and Y-SnO_2_ nanobelt in air at room temperature. It shows that the curves are nearly linear, revealing good Ohmic contacts between SnO_2_ NB and Y-SnO_2_ NB with the electrodes. The resistance of SnO_2_ NB is about 2.01 × 10^9^ Ω and that of Y-SnO_2_ NB is about 6.69 × 10^8^ Ω. The ratio of their resistance is about 3, which is much larger than that of their surface ratio. Therefore, the dopant improves the conductance of Y-SnO_2_ NB.

**Fig. 7:**
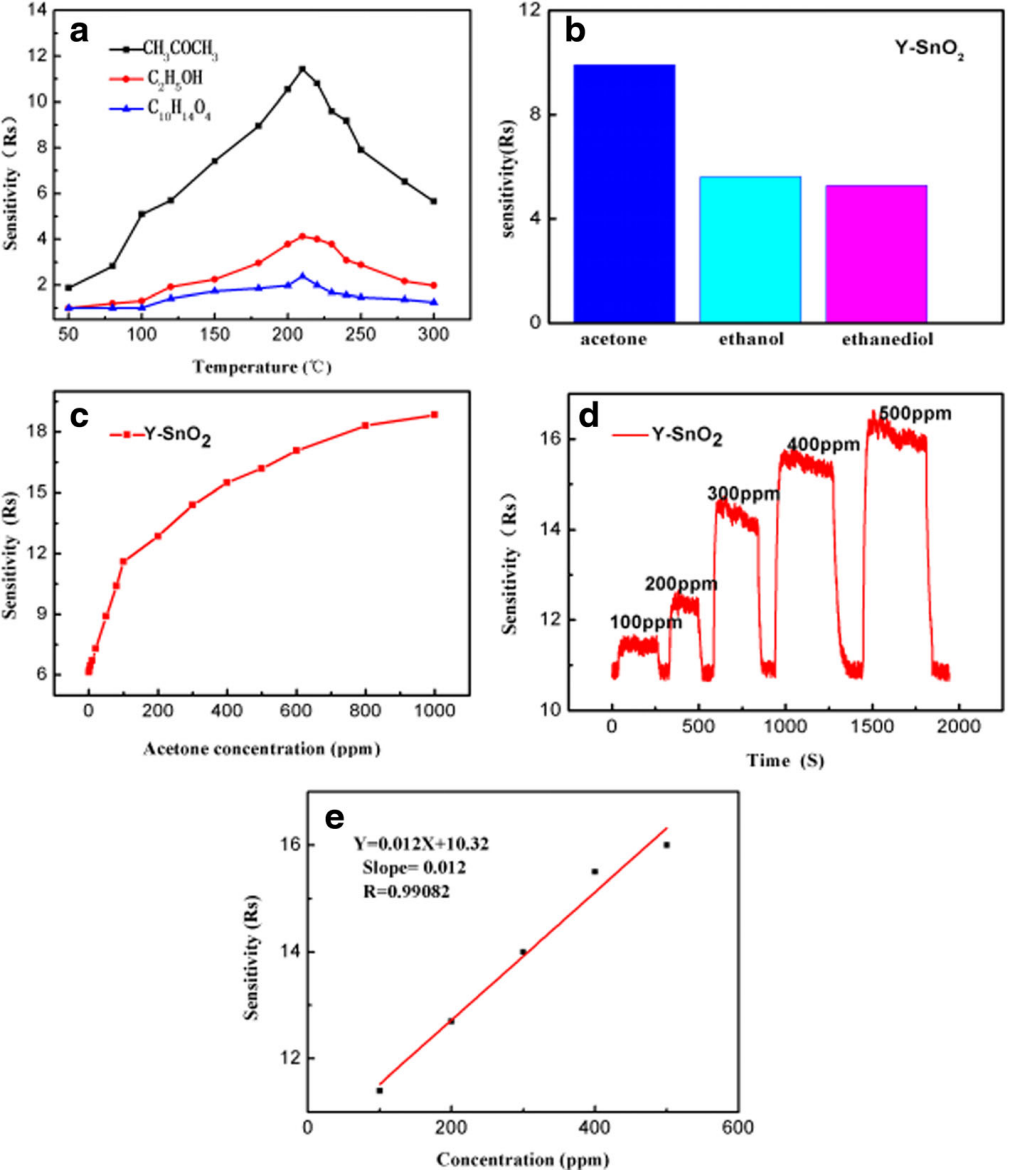
The sensitivity curves of the Y-SnO_2_ NB (**a**). The histogram of the Y-SnO_2_ NB device corresponding to 100 ppm of different gases at 210 °C (**b**). The response of the Y-SnO_2_ NB sensor is further investigated as a function of acetone gas concentration at 210 °C (**c**). The response (recovery) time of the Y-SnO_2_ NB device (**d**). Fitting the curve of response versus acetone concentration in the range of 100–500 ppm (**e**)

Figure 7a shows the sensitivity of the Y-SnO_2_ NB device as it is exposed to 100 ppm of ethanediol, ethanol, and acetone gases at different operating temperatures from 50 to 300 °C. It is noted that the sensitivity increases with an increment of the temperature up to 210 °C, and then starts to fall. Therefore, the optimum working temperature of sensor to ethanediol, ethanol, and acetone gases is 210 °C with a response of 11.4 to acetone. The histogram of the Y-SnO_2_ NB device corresponding to 100 ppm of different gases at 210 °C is shown in Fig. 7b. The response to 100 ppm of acetone is 11.4 at 210 °C, which is 2.7 times and 4.7 times as large as to ethanol and ethanediol, respectively. Under the condition of the same concentration of acetone, its response is 9.04 times as large as that of its pure counterpart. The result reveals that the response of Y-SnO_2_ NB sensor to acetone gas has good selectivity. Figure 7c shows that the response of Y-SnO_2_ NB sensor is further investigated as a function of acetone concentration at 210 °C. It is seen that the sensitivity increases with an increase of acetone concentration from 0 to 100 ppm, and then slowly becomes from 100 to 800 ppm, and finally nearly reaches a saturated state from 800 to 1000 ppm. It is also observed that the resistance of SnO_2_ NB declines significantly upon injection of acetone gas and returns to its original state when acetone vapor is expelled, as shown in Fig. 7d. The response (recovery) time is about 9–25 s/10–30 s to acetone at 210 °C. Repeated measurements have corroborated that the Y-SnO_2_ NB device possesses good selectivity and stability to acetone. Figure 7e shows the fitting curve of the sensitivity versus acetone concentration in 100–500 ppm. Its slope is 0.012 ppm^−1^ with a correlation coefficient *R* of 0.9908. One hundred forty data points in Fig. 7d at the baseline were selected to calculate a standard deviation (*S* = 0.0428). According to $$ {\mathrm{RMS}}_{\mathrm{noise}}=\sqrt{\raisebox{1ex}{${S}^2$}\!\left/ \!\raisebox{-1ex}{$N$}\right.} $$, the RMS_noise_ is 0.0036 for acetone sensor [24]. The detection limit can be written as DL (ppm) = 3 × RMS_noise_/slope, where 3 is the signal-to-noise ratio and RMS_noise_ represents the sensor noise [20]. Therefore, the detection limit of the sensor is 0.9024 ppm.

**Fig. 8:**
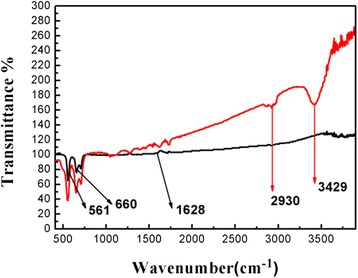
FTIR spectra of pure SnO_2_ NBs and Y-SnO_2_ NBs in the range of 400–4000 cm^−1^

The FTIR spectra of pure SnO_2_ NBs and Y-SnO_2_ NBs at room temperature are shown in Fig. 8. It is well known that FTIR is a powerful tool to identify functional groups or the types of chemical bonds. It is clearly seen that the peaks appear at around 561, 660, and 1628 cm^−1^ for pure SnO_2_. The spectrum of SnO_2_ NBs contains resonance stretching vibration modes in the range of 400–800 cm^−1^. The peaks at 561 and 660 cm^−1^ belong to the Sn–O stretching vibration modes [25, 26]. A weak peak at 1628 cm^−1^ is recognized as the deformation mode of OH groups [27]. We can also find that the increment of Y content causes a small shift to a lower wave number and the absorption peak becomes stronger. Some literatures have been reported to study the acetone absorption spectrum of gas sensor [28–30]. For example, Zhang et al. reported that the absorption peaks at 3582, 2968, 1731, 2929, and 1234 cm^−1^ were detected [28–30] as SnO_2_ sensor is exposed to acetone and then absorbs acetone vapor on its surface. Those vibration peaks could be assigned to the absorbed acetone *v*(OH), *v*(C–H), *v*(C=O), and *v*(C–C), respectively [28]. However, FTIR absorption peaks of Y-SnO_2_ NBs located at 2930 and 3429 cm^−1^ are close to the peak position of the absorbed acetone on the surface of the SnO_2_ centered at 2929 and 3582 cm^−1^, respectively. It shows that Y-SnO_2_ sensor easily absorbs acetone. Therefore, the Y-SnO_2_ NB sensor is sensitive to acetone, which supported the result of its sensing properties.

### Mechanism of the Sensitivity of Y-SnO_2_ NBs

The sensitivity of oxide (n-type semiconductor oxide) nanobelt depends on the barrier height of its grain boundary, grain size, lattice defects, the amount of oxygen absorption on the surface, catalyst crystallinity, etc [31–33]. A lot of oxygen molecules are absorbed on the surface of SnO_2_ NBs in air, resulting in the formation of a donor level and producing O^−^ or O^2−^ ions. The process is as follows:

- O_2_ (gas)→O_2_ (adsorption)
- O_2_ (gas)→O_2_^−^ (adsorption)
- O_2_^−^ (gas)→2O^−^ (adsorption)
- O_2_^−^ (gas)→O^2−^ (adsorption)

Nanometer sensor’s surface is negatively charged and then leads to the free electron concentration of SnO_2_ reduce so that the depletion layer is formed. The conductivity of metal oxide is dominated by the potential barrier formed at the grain boundaries. Reducing gas will react with the absorbed oxygen molecules when the gas sensor is exposed to the target gas so that released trapped electron will enter into the lattices of SnO_2_. Thus, the barrier height decreases and its conductivity increases. For organic volatile acetone, the reaction process is expressed as follows:

- 4O_2_^−^ + C_3_H_6_O = 3H_2_O + 3CO_2_ + 8e^−^
- 8O^−^ + C_3_H_6_O = 3H_2_O + 3CO_2_ + 8e^−^
- 8O^2−^ + C_3_H_6_O = 3H_2_O + 3CO_2_ + 16e^−^

**Fig. 9:**
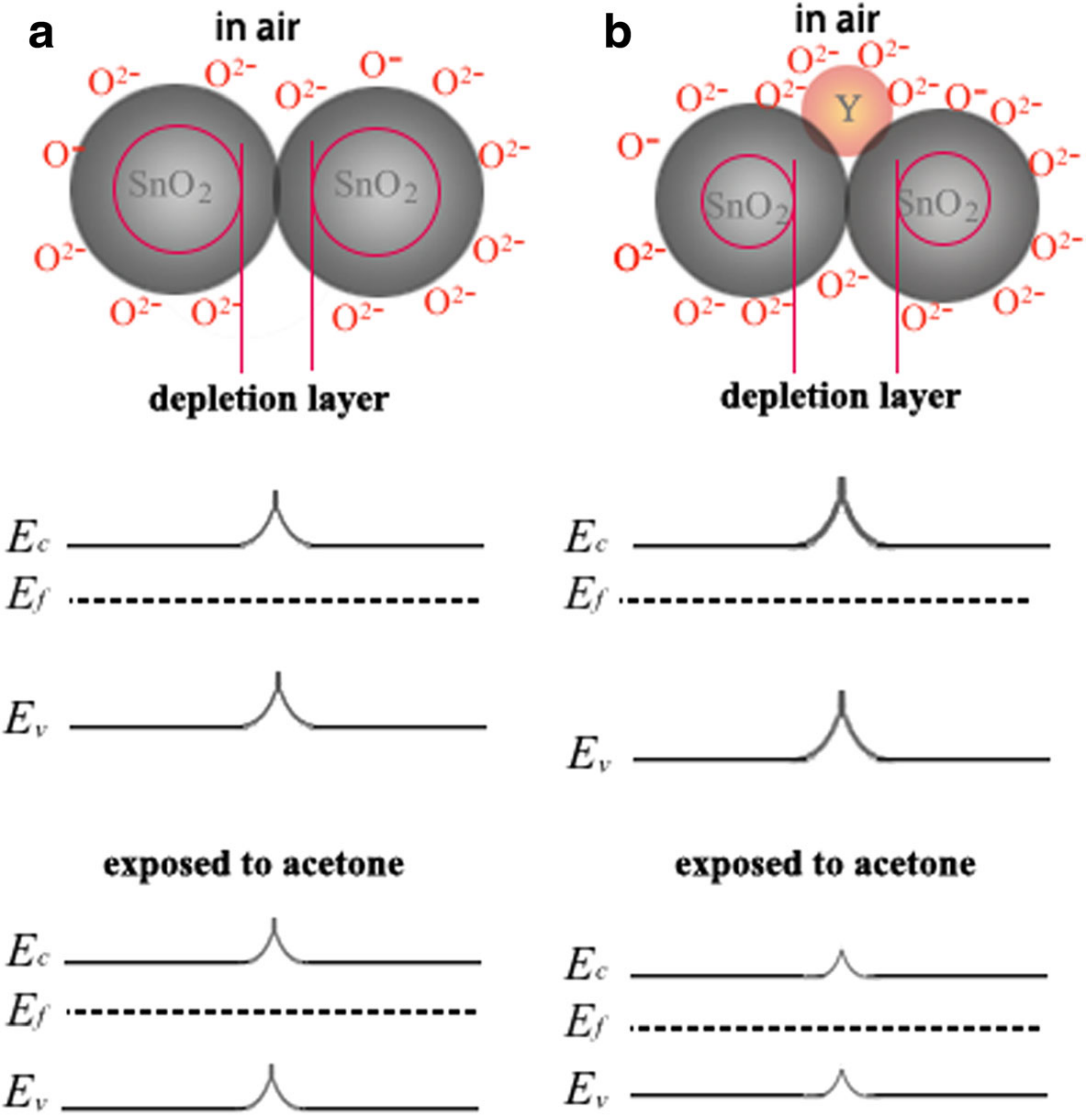
The sensing mechanism of Y-SnO_2_ NBs in the acetone environment (**a**, **b**)

On the other hand, it is well known that doping can also lower the barriers’ height and make the depletion layer thinner. These effects improve its electrical conductivity and enhance its sensing performances. The abovementioned mechanism can be depicted in Fig. 9.

## Conclusions

Y-SnO_2_ NBs have been synthesized by thermal evaporation method. The XRD pattern indicates that Y-SnO_2_ NBs and undoped counterparts are a tetragonal structure. The EDS and XPS results reveal that Y^3+^ ions are doped into SnO_2_ NBs successfully. Compared with that of pure SnO_2_, the UV-Vis absorption spectrum of Y-SnO_2_ NBs redshifts after doping. In addition, the sensing property of the device based on Y-SnO_2_ NB has been measured at different concentrations. It is found that the Y-SnO_2_ NB device have a higher sensitivity with 11.4 to 100 ppm of acetone at 210 °C and the doping of Y improves the sensing performance of SnO_2_ NBs effectively.

## Additional file

Additional file 1: Supporting information. (DOC 440 kb)
