# Supplementary material for: The Sensing Properties of Single Y-Doped SnO2 Nanobelt Device to Acetone
Source: Nanoscale Res Lett. 2016 Oct 21;11:470. doi: 10.1186/s11671-016-1685-1 (PMC5074997; doi:10.1186/s11671-016-1685-1)
Supplement: Additional file 1: — Supporting information. (DOC 440 kb) [file 11671_2016_1685_MOESM1_ESM.doc]

**Supporting Information**

Optical graphics of the single Y-SnO_2_ nanobelt device and its counterpart (pure SnO_2_ nanobelt device) were shown in Fig.S1 (a) and Fig. S1 (b). Their high magnification images were separately shown in Fig. S1 (c) and Fig. S1 (d). The two nanoribbons possess the same thickness of 50 nm. The surface ratio of the Y-SnO_2_ NB to the pure SnO_2_ NB is calculated as follows: for Y-SnO_2_ device, S _Y surface area_ = 10.35μm (length) × 0.5688μm (width)+ 0.05μm × 10.35μm × 2= 6.92208μm^2^ ; for its counterpart, S_surface area_= 10.36μm (length) × 0.4721μm (width)+ 0.05μm × 10.36μm × 2= 5.926956μm^2^. Therefore, the ratio of their surface areas is 1.1. However, the resistance of the pure SnO_2_ is about 2.01×10^9^ Ω and that of Y-SnO_2_ NB is about 6.69×10^8^ Ω. The ratio of their resistance is 3, which is much larger than that of their surface ratio. Therefore, the dopant improves the conductance of the Y-SnO_2_ NB.


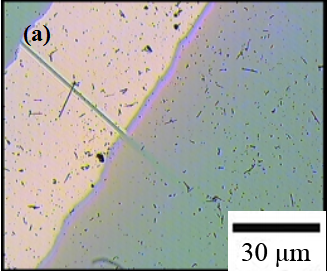

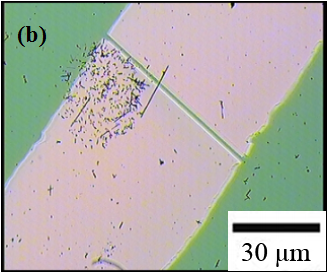


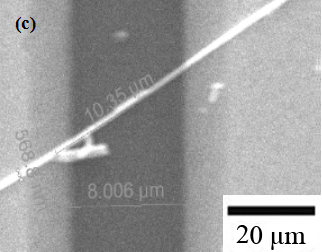

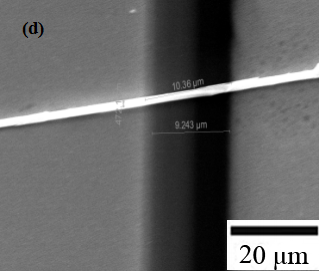


Fig.S1 Optical graphics and SEM images of the single Y-SnO_2_ nanobelt device and its counterpart

(a) (c) Y-SnO_2_ NB; (b)(d) SnO_2_ NB
